# GsMTx4 reduces the reflex pressor response during dynamic hindlimb skeletal muscle stretch in decerebrate rats

**DOI:** 10.14814/phy2.13974

**Published:** 2019-01-10

**Authors:** Bailey C. Sanderson, Korynne S. Rollins, Tyler D. Hopkins, Alec L. Butenas, Kennedy P. Felice, Carl J. Ade, Steven W. Copp

**Affiliations:** ^1^ Department of Kinesiology Kansas State University Manhattan Kansas

**Keywords:** Blood pressure, exercise pressor reflex, mechanoreflex, piezo channels

## Abstract

Mechanical signals within contracting skeletal muscles contribute to the generation of the exercise pressor reflex; an important autonomic and cardiovascular control mechanism. In decerebrate rats, the mechanically activated channel inhibitor GsMTx4 was found to reduce the pressor response during static hindlimb muscle stretch; a maneuver used to investigate specifically the mechanical component of the exercise pressor reflex (i.e., the mechanoreflex). However, the effect was found only during the initial phase of the stretch when muscle length was changing and not during the later phase of stretch when muscle length was relatively constant. We tested the hypothesis that in decerebrate, unanesthetized rats, GsMTx4 would reduce the pressor response throughout the duration of a 30 sec, 1 Hz dynamic hindlimb muscle stretch protocol that produced repetitive changes in muscle length. We found that the injection of 10 *μ*g of GsMTx4 into the arterial supply of a hindlimb reduced the peak pressor response (control: 15 ± 4, GsMTx4: 5 ± 2 mmHg, *P* < 0.05, *n* = 8) and the pressor response at multiple time points throughout the duration of the stretch. GsMTx4 had no effect on the pressor response to the hindlimb arterial injection of lactic acid which indicates the lack of local off‐target effects. Combined with the recent finding that GsMTx4 reduced the pressor response only initially during static stretch in decerebrate rats, the present findings suggest that GsMTx4‐sensitive channels respond primarily to mechanical signals associated with changes in muscle length. The findings add to our currently limited understanding of the channels that contribute to the activation of the mechanoreflex.

## Introduction

The exercise pressor reflex is activated when mechanical and metabolic signals arising from within contracting skeletal muscles stimulate the sensory endings of group III and group IV muscle afferents (Mitchell et al. 1983). Those feedback signals are integrated in the medulla of the brainstem and contribute to increases in sympathetic nervous system activity, heart rate, and blood pressure which facilitates increased perfusion of contracting skeletal muscles (O'Leary et al. 1999; Amann et al. 2011). The reflex was classically considered to be an ischemically activated reflex (Rowell 1993) which has resulted in a long‐standing interest in the reflex's metabolically sensitive component (i.e., the metaboreflex). More recently, interest has increased in the reflex's mechanically sensitive component (i.e., the mechanoreflex). This interest has been driven, in part, by findings that mechanoreflex alterations contribute to the exaggerated exercise pressor reflex present in multiple forms of cardiovascular disease (Middlekauff et al. 2001, 2004; Smith et al. 2005; Leal et al. 2008; Muller et al. 2012, 2015; Lu et al. 2013).

The mechanoreflex may be studied in humans and animals by investigating the autonomic and/or cardiovascular responses to passive movement or stretch of limb skeletal muscles (e.g., Tibes 1977; Stebbins et al. 1988; Smith et al. 2001; Middlekauff and Chiu 2004; Fisher et al. 2005; Cui et al. 2006; Ives et al. 2016; Drew et al. 2017). In decerebrate, unanesthetized rats, Copp et al. (2016a) recently investigated the effect of GsMTx4, a tarantula peptide that inhibits mechanically activated channels (Bae et al. 2011), on the mechanoreflex evoked by static hindlimb muscle stretch and the exercise pressor reflex evoked by dynamic hindlimb muscle contraction. GsMTx4 reduced the pressor response during both static stretch and dynamic contraction (Copp et al. 2016a). However, the effect of GsMTx4 on the pressor response during static stretch was found only during the first ~5 sec of the 30‐sec protocol when muscle length was changing whereas there was no effect on the pressor response during the sustained phase when muscle length was relatively constant. In contrast, GsMTx4 reduced the pressor response throughout the duration of the 30‐sec dynamic contraction protocol in which muscle length changed repetitively (Copp et al. 2016a). The mechanistic underpinnings of the sustained effect of GsMTx4 throughout dynamic contraction compared to the only transient effect of GsMTx4 during static stretch are unknown. The two most likely possibilities are that GsMTx4‐sensitive channels respond primarily to changes in muscle length or that metabolic signals associated with dynamic contraction sensitized GsMTx4‐sensitive channels which prolonged their stimulation throughout the contraction maneuver. Investigating those possibilities would contribute to our currently limited understanding of the mechanically activated channels that underlie the mechanoreflex and the exercise pressor reflex across different modalities.

The purpose of this investigation was to experimentally separate repetitive changes in muscle length from contraction‐induced metabolite production in order to shed further light on the properties of the GsMTx4‐sensitive channels associated with the mechanoreflex and the exercise pressor reflex. Specifically, we tested the hypothesis that in decerebrate, unanesthetized rats, GsMTx4 would reduce the pressor response throughout the duration of a 1 Hz dynamic hindlimb skeletal muscle stretch protocol. We recently used such a protocol as an experimental tool to investigate the mechanoreflex signals present during 1 Hz dynamic hindlimb skeletal muscle contraction in decerebrate rats (Kempf et al. 2018).

## Material and Methods

All experimental procedures were approved by the Institutional Animal Care and Use Committee of Kansas State University and were conducted in accordance with the National Institutes of Health Guide for the Care and Use of Laboratory Animals. Experiments were performed on young adult male Sprague‐Dawley rats (*n* = 17, average body weight: 385 ± 10 g). The rats were housed two per cage in temperature‐ (maintained at ~22°C) and light‐(12/12 h light/dark cycle running from 7 am to 7 pm) controlled accredited facilities with standard rat chow and water provided ad libitum. At the end of each experiment, the decerebrated rats (see below) were euthanized with an intravenous (i.v.) injection of saturated (>3 mg/kg) potassium chloride.

### Surgical procedures

All rats were anesthetized initially with 5% isoflurane (balance O_2_). Depth of anesthesia was confirmed by an absent toe‐pinch reflex. A tracheostomy was performed, and rats’ lungs were mechanically ventilated (Harvard Apparatus) with gaseous anesthetic (2% isoflurane balance O_2_). The right jugular vein and both common carotid arteries were cannulated with PE‐50 catheters for administration of drugs, measurement of arterial blood pressure (Physiological Pressure Transducer, AD Instruments), and sampling of arterial blood gases. In 12 rats, a reversible snare (2–0 suture) was placed around the left common iliac artery and vein just distal to the descending aorta/inferior vena cava and the left superficial epigastric artery was cannulated with a PE‐8 catheter for administration of drugs. In all rats, the left calcaneus bone was severed and the triceps surae (gastrocnemius, soleus, and plantaris) muscles were exposed by carefully removing the overlying skin and skeletal muscle. A string was then tied to the distal Achilles tendon and severed calcaneus which was used to link the triceps surae muscles to a force transducer (Grass FT03) which was attached to a rack and pinion that could be manually turned.

Upon completion of the initial surgical procedures, all rats were secured in a Kopf stereotaxic frame. After administering dexamethasone (0.2 mg i.v.) to minimize swelling of the brainstem, a decerebration was performed in which all brain tissue rostral to the superior colliculi was removed (Smith et al. 2001). Following decerebration, anesthesia was terminated, and the rats’ lungs were ventilated with room air. All rats were allowed a minimum of 60 min to recover from anesthesia before initiation of any experimental protocol. Experiments were performed on decerebrate, unanesthetized rats because anesthesia has been shown to markedly blunt the mechanoreflex and exercise pressor reflex in the rat (Smith et al. 2001). Body core temperature was measured via rectal probe and maintained at ~37–38°C by an automated heating system (Harvard Apparatus) and heat lamp. Arterial pH and blood gases were analyzed periodically throughout the experiment from small blood samples (~75 *μ*L) and maintained within physiological ranges (pH: 7.35–7.45; pCO_2_: ~38–40 mmHg; PO_2_: ~100 mmHg) by administration of sodium bicarbonate and/or adjusting ventilation. Prior to the initiation of the experimental protocol, each rat was paralyzed with injection of pancuronium bromide (~1 mg/kg i.v.).

### Primary experimental protocol

In eight rats, we compared the increase in blood pressure during a 1 Hz dynamic stretch protocol of the triceps surae muscles and following injection of 24 mmol/L lactic acid before and after intra‐arterial injection of GsMTx4 into the hindlimb circulation. In detail, following the minimum 60 min post decerebration recovery period, baseline muscle tension was set at ~100 g by manually turning the rack and pinion. Blood pressure and heart rate were collected for ~30 sec. A dynamic triceps surae muscle stretch protocol was then initiated by an experienced investigator in which the rack and pinion were turned back and forth manually at a 1 Hz frequency with the aid of a metronome for at least 30 sec. The investigator aimed for a tension generation of ~0.7–0.8 kg on each stretch with the length of time of each tension development being ~0.5 sec. This protocol was adapted from that described by Daniels et al. (2000) in the cat. We used this protocol recently to investigate mechanoreflex activation during dynamic stretch in decerebrate rats (Kempf et al. 2018). Approximately 5 min following the completion of the first (i.e., control) dynamic stretch protocol and after ensuring that blood pressure had returned to its prestretch baseline value, we injected lactic acid (0.2 mL of 24 mmol/L concentration in saline) into the arterial supply of the hindlimb via the superficial epigastric artery catheter. After another approximately 5‐min waiting period and after ensuring blood pressure once again returned to baseline, we tightened the iliac snare and injected GsMTx4 (10 *μ*g dissolved in 0.2 mL of saline) into the arterial supply of the hindlimb via the superficial epigastric artery catheter. The toxin remained snared in the hindlimb circulation for 10 min, at which time the iliac snare was released and the leg reperfused for 20 min. The dose of GsMTx4 and injection protocol were identical to that used recently by Copp et al. (2016a) to investigate the effect of GsMTx4 on the pressor response during static hindlimb muscle stretch and dynamic hindlimb muscle contraction in decerebrate rats. After the 20‐min reperfusion period, the dynamic stretch and lactic acid injection protocols were repeated exactly as described above. The tension generated during the post‐GsMTx4 stretch was matched as closely as possible to that produced during the pre‐GsMTx4 control stretch. Evans blue dye was then injected into the arterial supply of the hindlimb in the same manner that GsMTx4 was injected to ensure that the toxin had access to the triceps surae muscle circulation in all experiments. The triceps surae muscles were observed to stain blue in all experiments.

### Control experimental protocols

In an additional group of five rats, we compared the pressor response during 30 sec of 1 Hz dynamic stretch before and after i.v. administration of GsMTx4. Specifically, following the 60 min post decerebration recovery period, we performed the dynamic stretch protocol exactly as described above for the primary experimental protocol. Following a ~5 minute recovery period, GsMTx4 (10 *μ*g dissolved in 0.2 mL of saline) was injected into the right jugular vein catheter. Thirty minutes following the i.v. injection of GsMTx4, the dynamic stretch protocol was repeated.

In another group of four rats, we compared the pressor response during dynamic stretch before and after intra‐arterial administration of saline, the vehicle for GsMTx4. Specifically, following the 60 min post decerebration recovery period, we performed the dynamic stretch protocol exactly as described above for the primary experimental protocol. Following a 5 min recovery period, the iliac snare was tightened, and 0.2 mL of saline was injected into the arterial supply of the hindlimb via the superficial epigastric artery catheter. The snare was released 10 min following the injection of saline, and the dynamic stretch protocol was repeated 20 min following the release of the snare.

### Data and statistical analysis

Arterial blood pressure and muscle tension were measured (PowerLab and LabChart data acquisition; AD Instruments) and mean arterial pressure (MAP) and heart rate (HR) were calculated in real time and recorded for offline analysis. Baseline MAP and HR were determined for the 30‐sec baseline periods that preceded each maneuver (stretch or lactic acid injection). The time‐independent peak increase in MAP (peak Δ MAP) and HR (peak Δ HR) during dynamic stretch or lactic acid injection was calculated as the difference between the peak values wherever they occurred during the maneuvers and the corresponding baseline value. The time course of the increase in MAP was plotted as the Δ MAP from baseline over the course of the 30‐sec stretch protocols. The change in the tension‐time‐integral (Δ TTI) during stretch above baseline was calculated by integrating the area under the tension signal and subtracting the integrated area during the baseline period.

Data are expressed as mean ± SEM. Data were compared with paired Student's *t*‐tests or repeated measures ANOVAs with Holm–Sidak post hoc tests as appropriate. Statistical significance was defined as *P* < 0.05.

## Results

**Figure 1:**
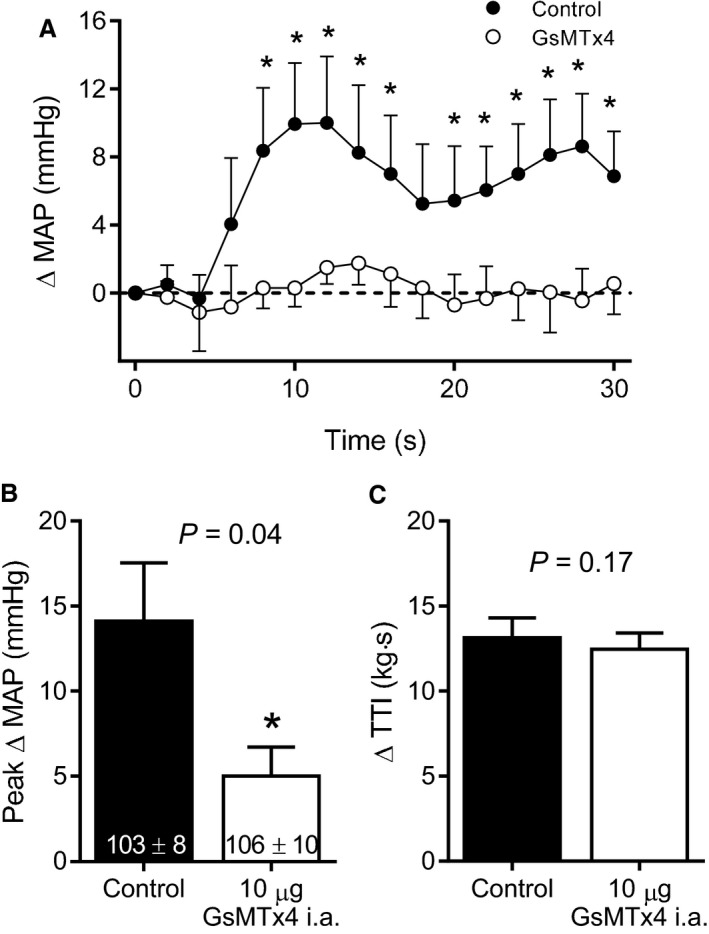
Injection of 10 *μ*g of GsMTx4 into the arterial supply of the hindlimb (*n* = 8) significantly reduced the pressor response at multiple time points throughout the duration of 1 Hz dynamic hindlimb muscle stretch (A). GsMTx4 also reduced the time‐independent peak pressor response during stretch (B). Baseline blood pressure values are shown within the respective mean bars in panel B and were not significantly different between conditions. MAP = mean arterial pressure, TTI = tension‐time‐integral. Asterisks indicate statistically significant differences (*P* < 0.05) compared to control. The horizontal dashed line is representative of the baseline blood pressure.

**Figure 2:**
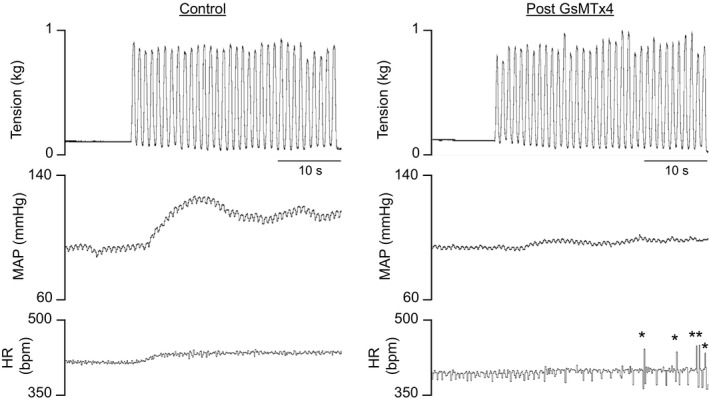
An example of an original recording showing tension developed, mean arterial pressure (MAP), and heart rate (HR) during dynamic stretch before (control) and after the hindlimb arterial injection on 10 *μ*g of GsMTx4. Asterisks indicate artifacts in the HR signal.

**Figure 3:**
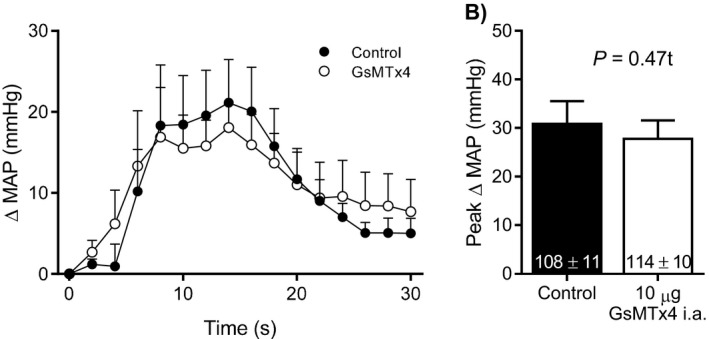
Injection of 10 *μ*g of GsMTx4 into the arterial supply of the hindlimb (*n* = 8) had no effect on the pressor response to the injection of lactic acid (24 mmol/L, 0.2 mL) into the arterial supply of the hindlimb, indicating that GsMTx4 did not exert off‐target effects. Baseline blood pressure values are shown within the respective mean bars in panel B and were not significantly different between conditions. MAP = mean arterial pressure.

Thirty seconds of 1 Hz dynamic hindlimb muscle stretch markedly increased MAP and HR. The peak increase in MAP occurred, on average, 9 sec following the onset of stretch and remained elevated above baseline for the duration of the maneuver. In eight rats, the injection of GsMTx4 into the arterial supply of the hindlimb significantly reduced the pressor response during dynamic stretch; an effect that was evident at multiple time points throughout the duration of the maneuver (Fig. 1A, see example of original tracing in Fig. 2). Moreover, compared to control, GsMTx4 significantly reduced the peak pressor response during dynamic stretch (Fig. 1B) whereas the effect on peak Δ HR did not reach statistical significance (control: 11 ± 2, GsMTx4: 6 ± 2 bpm, *P* = 0.07). GsMTx4 had no effect on baseline HR (control: 468 ± 18, GsMTx4: 456 ± 18 bpm, *P* = 0.40). There were no differences in the Δ TTI (Fig. 1C) or the average peak Δ tension (control: 0.73 ± 0.05, GsMTx4: 0.72 ± 0.04 kg, *P* = 0.20) between control and GsMTx4 conditions. In these same rats in which we performed dynamic stretch, we also injected lactic acid into the arterial supply of the hindlimb before and after the injection of GsMTx4. We found that GsMTx4 had no effect on the time course of the pressor response or the peak pressor response that resulted from the injection of lactic acid (Fig. 3) which is consistent with the notion that GsMTx4 did not have off‐target effects on skeletal muscle sensory neurons such as the inhibition of voltage‐gated sodium (NaV) channels (Redaelli et al. 2010).

**Figure 4:**
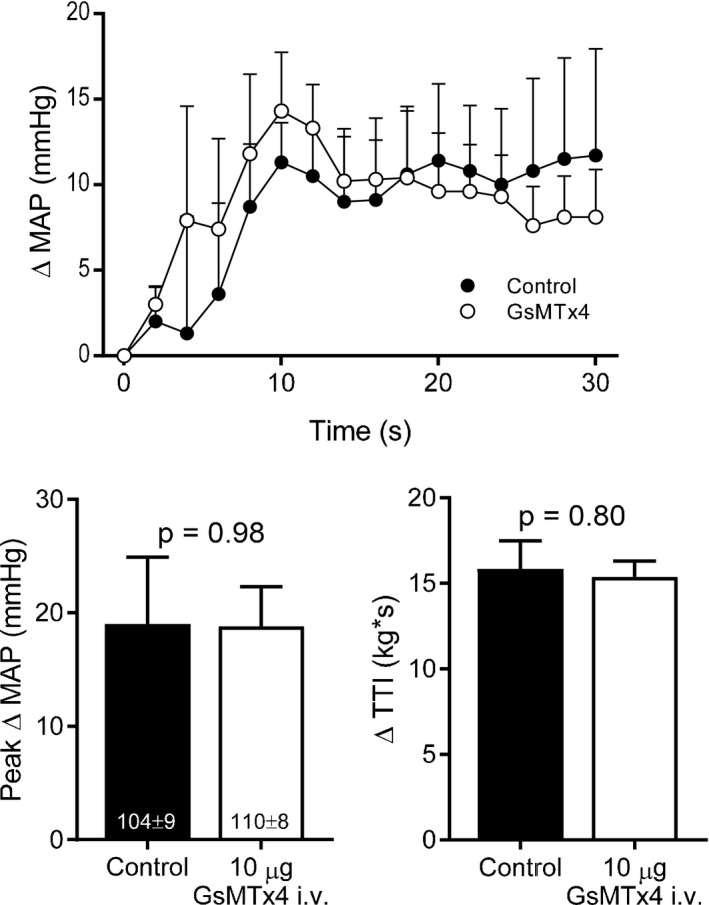
Injection of 10 *μ*g of GsMTx4 into the jugular vein (*n* = 5) had no effect on the time course of the pressor response (A) or the time‐independent peak pressor response (B) during 1 Hz dynamic hindlimb skeletal muscle stretch. Baseline blood pressure values are shown within the respective mean bars in panel B and were not significantly different between conditions. MAP = mean arterial pressure, TTI = tension‐time‐integral.

In an additional group of five rats, we compared the pressor response during dynamic stretch before and after the injection of 10 *μ*g of GsMTx4 into the jugular vein to determine if the effects observed when injected into the hindlimb arterial supply could be attributed to systemic effects elsewhere in the mechanoreflex arc such as the spinal cord or the medulla of the brainstem, for example. The injection of GsMTx4 into the jugular vein had no effect on the time course of the pressor response (Fig. 4A), the peak pressor response (Fig. 4B), or the peak HR response (control: 12 ± 6, GsMTx4: 10 ± 3 bpm, *P* = 0.79) during dynamic stretch. GsMTx4 also had no effect on baseline HR (control: 522 ± 22, GsMTx4: 549 ± 43 bpm, *P* = 0.48). There were no differences in the Δ TTI (Fig. 4C) or the average peak Δ tension (control: 0.81 ± 0.07, GsMTx4: 0.83 ± 0.06 kg, *P* = 0.08) of the stretches for the i.v. injection control experiments. Likewise, in another group of four rats, the injection of saline (the vehicle for GsMTx4) into the arterial supply of the hindlimb had no effect on the time course of the pressor response (data not shown), the peak pressor response (control: 18 ± 4, GsMTx4: 18 ± 2 mmHg, *P* = 0.89), or the peak HR response (control: 13 ± 5, GsMTx4: 9 ± 3 bpm, *P* = 0.17) during dynamic stretch. Saline had no effect on baseline HR (control: 482 ± 21, saline: 491 ± 21 bpm, *P* = 0.68). There were no differences in the Δ TTI (control: 14.3 ± 1.3, saline: 15.3 ± 1.8 kg·sec, *P* = 0.18) or the average peak Δ tension (control: 0.72 ± 0.07, GsMTx4: 0.72 ± 0.07 kg, *P* = 0.80) of the stretches between conditions for the saline control experiments.

## Discussion

Consistent with our hypothesis, we found that the hindlimb arterial injection of GsMTx4 in decerebrate rats reduced the pressor response throughout the duration of a 1 Hz dynamic hindlimb skeletal muscle stretch protocol. The present finding contributes to our understanding of the channels that underlie the mechanical component of the exercise pressor reflex during dynamic contractions. Specifically, in healthy decerebrate rats, GsMTx4‐sensitive channels appear to respond primarily to changes in muscle length. By implication, exercise/contraction protocols that produce repetitive changes in muscle length are likely to produce a significant stimulus to GsMTx4‐sensitive channels.

In the present investigation, we found that the hindlimb arterial injection of GsMTx4 had no effect on the pressor response that resulted from the injection of lactic acid. As discussed in detail recently (Copp et al. 2016a), if GsMTx4 had inhibited NaV channels on the axons of the muscle afferents (Redaelli et al. 2010) we would have expected the toxin to reduce the pressor response that resulted from the injection of lactic acid. We also found that the injection of GsMTx4 into the jugular vein had no effect on the pressor response during dynamic stretch. If GsMTx4 had produced a nonspecific effect in the spinal cord or brainstem we would have expected i.v. injection of the toxin to reduce the pressor response during dynamic stretch. In our final set of control experiments, we found that the hindlimb arterial injection of saline had no effect on the pressor response during dynamic stretch which served as a time and vehicle control. Collectively, therefore, the present findings suggest that the hindlimb arterial injection of GsMTx4 reduced the pressor response throughout dynamic stretch by inhibiting mechanically activated channels on the sensory endings of thin fiber muscle afferents.

Our present finding, and the conclusion that GsMTx4‐sensitive channels respond primarily to changes in muscle length, is consistent with our recent finding that GsMTx4 reduced the pressor response throughout dynamic contraction but only initially during static stretch in normal rats with patent femoral arteries (Copp et al. 2016a). In addition to stimulating GsMTx4‐sensitive channels initially, static hindlimb muscle stretch likely stimulated different mechanically sensitive channels that responded primarily to the level of tension development during the sustained phase of the stretch. In contrast to our recent findings in rats with patent femoral arteries (Copp et al. 2016a), in a rat model of simulated peripheral artery disease in which a femoral artery is chronically ligated, we found that GsMTx4 reduced the pressor response throughout both dynamic contraction and static stretch (Copp et al. 2016b). Thus, the conclusion of the present investigation that GsMTx4‐sensitive channels respond primarily to changes in muscle length in normal rats with patent femoral arteries does not appear to extend to the ligated rat model of simulated peripheral artery disease. The reason for this is unknown but we speculate that a chronic metabolite/chemical sensitization of GsMTx4‐sensitive channels may have occurred in “ligated” rats such that channel activity was prolonged throughout static stretch.

Nakamoto and Matsukawa (2007) demonstrated the possibility of different anatomical locations of the mechanically sensitive channels that respond to changes in muscle length compared to those that respond to tension development. Specifically, the injection of lidocaine into the rat Achilles myotendinous junction to reduce afferent transmission reduced the pressor response during the sustained phase of static muscle stretch but not during the initial phase when muscle length changed rapidly. The implication was that channels that responded to changes in muscle length were located more centrally within muscles and channels that responded to muscle tension development were located near myotendinous junctions (Nakamoto and Matsukawa 2007). Considered together with our recent (Copp et al. 2016a) and present findings, GsMTx4‐sensitive channels may be located more centrally within rat hindlimb skeletal muscles. As suggested by Nakamoto and Matsukawa (2007), the concept that some mechanically activated channels may respond to different mechanical signals (i.e., changes in muscle length vs. muscle tension) is consistent with the finding that some group III muscle afferents (which tend to be primarily mechanically sensitive) in the cat displayed a rapidly adapting firing pattern at the onset of static hindlimb muscle contraction whereas others demonstrated a more sustained firing pattern (Kaufman et al. 1983).

In human subjects, the physiological importance of the mechanoreflex has been demonstrated by the autonomic and cardiovascular adjustments evoked by static or dynamic passive limb movement or muscle stretch (Gladwell and Coote 2002; Middlekauff and Chiu 2004; Fisher et al. 2005; Cui et al. 2006; Ives et al. 2013, 2016; Drew et al. 2017; Venturelli et al. 2017; Kruse et al. 2018). In human subjects, however, experimental limitations have hindered investigation of the channels that generate the reflex. In animal models, gadolinium has been the primary pharmacological tool used to inhibit the mechanically activated channels associated with the mechanoreflex and the exercise pressor reflex (Hayes and Kaufman 2001; Smith et al. 2005; Matsukawa et al. 2006; Nakamoto and Matsukawa 2007; Hayes et al. 2009; Mizuno et al. 2011). GsMTx4, on the other hand, has been used for that purpose only recently (Copp et al. 2016a,b). GsMTx4 has several advantages over gadolinium which includes the fact that GsMTx4 demonstrated at least some selectivity for specific classes of mechanically activated channels over others (Bae et al. 2011). Specifically, GsMTx4 was found to inhibit piezo 1 (Bae et al. 2011) and piezo 2 (Alcaino et al. 2017) channels but not the potassium selective mechanically activated channel TREK‐1 (Bae et al. 2011). Because of these facts, we believe piezo channels, especially piezo 2 channels, are likely to play a role in the mechanoreflex. GsMTx4, however, has also been shown to inhibit other classes of channels that demonstrate mechanical sensitivity including TRPC1 (Louis et al. 2008) and TRPC6 (Anderson et al. 2013) and the recently named and characterized tentonin 3 channel (Hong et al. 2016). Thus, although the present data may reflect GsMTx4‐mediated piezo channel inhibition given that they are highly expressed in dorsal root ganglia (Coste et al. 2010; Copp et al. 2016a) and demonstrate clear and robust mechanical sensitivity (Coste et al. 2010), we cannot rule out the involvement of other classes of mechanically activated channels.

### Experimental considerations

In rats, 87% of the muscle afferents that were found to respond to muscle stretch were also found to respond to muscle contraction (Stone et al. 2015). Based on that finding, we believe that the afferents stimulated by muscle stretch in our experiments would likely have also been stimulated by muscle contraction. Nevertheless, a limitation of the rat hindlimb muscle stretch model of mechanoreflex activation is that the afferents were stimulated by lengthening the muscle which was facilitated by cutting the calcaneus. During concentric contractions, mechanically sensitive afferents are stimulated when skeletal muscles shorten and develop tension (Victor et al. 1989; Copp et al. 2016a; Kempf et al. 2018). An advantage of the model used presently, however, is that mechanoreflex activation produces robust increases in sympathetic nerve activity and blood pressure similar to the increases found during rat hindlimb muscle contraction (Kempf et al. 2018).

Variability in the effect of GsMTx4 on the cardioaccelerator response during dynamic stretch in the present investigation resulted in the analysis of that effect not reaching statistical significance (*P* = 0.07). Conversely, in our recent experiments, GsMTx4 significantly reduced the cardioaccelerator response during static stretch (Copp et al. 2016a). The cardioaccelerator response during dynamic stretch represented only a ~2% increase in HR. Based on that fact, and because the effect of GsMTx4 on the pressor response during dynamic stretch was the primary study end point, we did not perform additional experiments to improve the statistical power of the cardioaccelerator analysis and we have focused our manuscript on the effect of GsMTx4 on the pressor component of the mechanoreflex.

### Summary and conclusion

We found that GsMTx4 reduced the pressor response throughout the duration of a 30 sec, 1 Hz dynamic stretch protocol in decerebrate rats. The present findings are an important extension of the recent finding that GsMTx4 reduced the pressor response only during the initial phase of static stretch but throughout dynamic contraction in decerebrate rats (Copp et al. 2016a). Specifically, the implication of the present findings are that in decerebrate rats with patent femoral arteries, GsMTx4‐sensitive channels associated with the mechanoreflex appear to respond primarily to changes in muscle length. By extension, the data suggest that differences may exist regarding the contribution of the class(es) of mechanically sensitive channels that underlie dynamic compared to static mechanoreflex and exercise pressor reflex modalities.

## Conflict of Interest

None.
